# Stepped waveguide metamaterials as low-loss effective replica of surface plasmon polaritons

**DOI:** 10.1515/nanoph-2022-0810

**Published:** 2023-03-01

**Authors:** Xu Qin, Yijing He, Wangyu Sun, Pengyu Fu, Shuyu Wang, Ziheng Zhou, Yue Li

**Affiliations:** Department of Electronic Engineering, Tsinghua University, Beijing 100084, China; School of Integrated Circuits and Electronics, Beijing Institute of Technology, Beijing 100081, China; College of Physics and Information Engineering, Fuzhou University, Fuzhou 350108, China; Beijing National Research Center for Information Science and Technology, Beijing 100084, China

**Keywords:** low-loss, plasmonics, stepped waveguide metamaterial, surface plasmon polaritons

## Abstract

Surface plasmon polaritons (SPPs) have attracted intensive attention for the unprecedented developments of light–matter interactions in optics and photonics, providing a feasible method for light confinement and transmission at a subwavelength scale. However, SPPs traditionally suffer from large losses due to the intrinsic dissipations and absorptions, which hinder further development and applications of SPPs. Here, we theoretically and experimentally investigate the concept of stepped waveguide metamaterials behaving as low-loss effective replicas of SPPs. The proposed structure without natural plasmonic material maintains the identical field configuration to that in regular SPP but avoids the inherent losses, outperforming regular low-loss SPP design with natural plasmonic materials on SPP propagation lengths. Furthermore, stepped waveguide metamaterial exhibits excellent compatibility in direct interconnections with arbitrary regular SPP and potentially represents a feasible route toward new SPP devices with low-loss advantages.

## 1 Introduction

Since Ritchie first investigated the fundamental physics of the surface electron oscillations [1], the light–matter interactions in optics and nanophotonics have been stepping into a new stage by surface plasmon polaritons (SPPs) on the interfaces between plasmonic materials and insulators over the past decades. Research based on SPPs has led to significant advances in light tailoring and manipulation at subwavelength scales, showing promising prospects for versatile optical and nanophotonic applications such as surface-enhanced spectroscopy [2–4], superresolution imaging and superfocusing [5–8], photonic circuits [9–12], and optical communications [13–16]. Additionally, the strong field-confinement effect enabled by SPP can significantly enhance the light–matter interactions in nanoscales, which fueled the advanced applications in biomedical imaging and sensing [17–19] and even facilitated quantum applications such as quantum information processing and quantum logic gates [20–22].

However, the SPPs have long been known to suffer from the inherent dissipations and absorptions in natural plasmonic materials [23], which has been a bottleneck of the further applications of SPP as well as plasmonics. The losses of plasmonic materials would have negative effects on the propagating length of SPPs as well as the sensitivity of SPP-based detecting devices [24]. Although suppressing the loss in the natural plasmonic materials can be a grand challenge [25–27], the rise of artificial structure-emulated SPPs can provide a promising solution [28]. In the latest research, artificial metamaterials with periodical structures, such as holes, grooves, slits, and blocks, have been proposed to achieve SPP-like fields in microwave frequency [29]. This new concept is named spoof SPP and has been successful in realizing low-loss SPPs at microwave frequencies where there are no traditional plasmonic materials. However, most research on spoof SPP has not focused on cooperating with existing regular SPP with natural plasmonic materials at optical frequencies.

**Figure 1:**
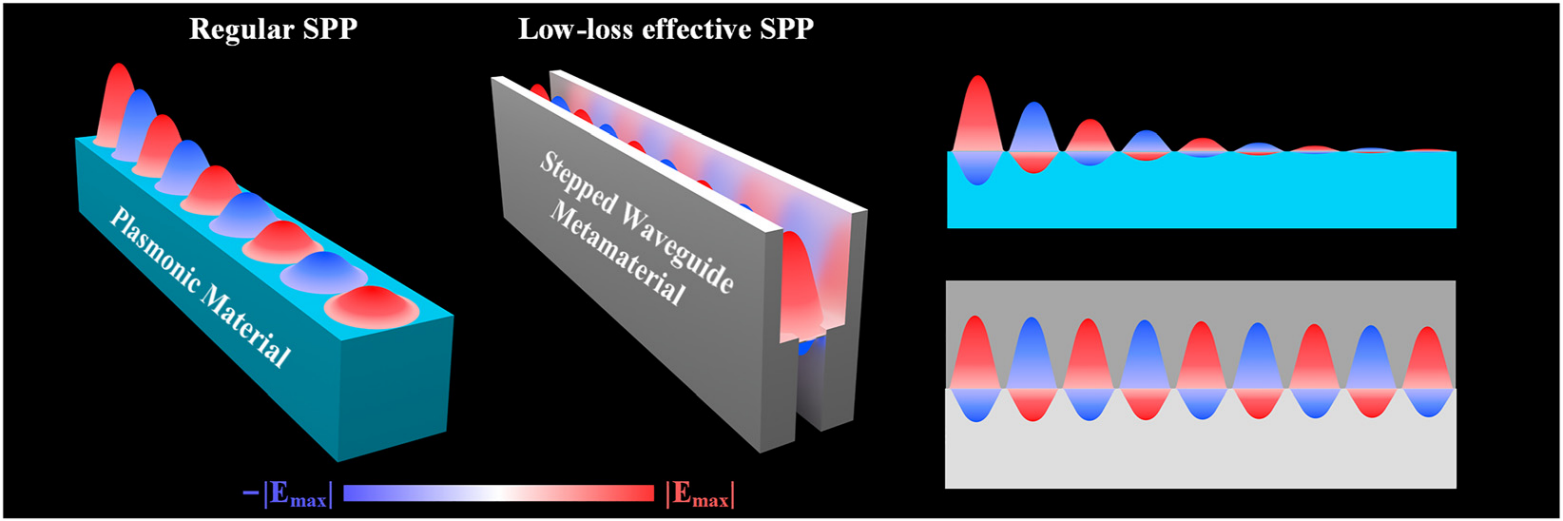
Concept of the stepped waveguide metamaterial. Conceptual sketches that show losses comparisons between regular SPP and stepped waveguide SPP.

Here, we propose a structure-based approach to realize a low-loss effective replica of an SPP without natural plasmonic materials, termed as “stepped waveguide metamaterial,” as shown in Figure 1. In this structure, we take advantage of the structural dispersion in guided-wave propagation to achieve a uniform effective plasmonic metamaterial [30]. Based on the structural dispersion of the stepped waveguide metamaterial, we can realize identical plasmonic properties and identical field distributions to a regular SPP supported by natural plasmonic materials, which is distinguished from normal spoof SPP and implies great compatibility with regular SPP. This method has been validated by both numerical analysis and experiments. Without natural plasmonic materials, the stepped waveguide metamaterial guarantees a low-loss effective surface plasmon wave operating up to near-infrared regions with the propagation length improved by 1–2 orders of magnitude compared to a regular SPP. The identical field distributions lead to excellent compatibility between a stepped waveguide SPP and regular SPP, and we exhibit a feasible interconnection and interaction between them. The stepped waveguide metamaterials avoid the material limit and could realize arbitrary plasmonic material properties. Moreover, based on the fundamental low-loss surface plasmon wave realized by the stepped waveguide metamaterial, various low-loss SPP applications could be achieved. As an example, the design of a symmetric stepped waveguide is proposed to realize an SPP propagation length larger than regular low-loss SPP waveguide design with traditional plasmonic materials [31–35], promising potential use in low-loss plasmonic devices.

## 2 Results

### 2.1 Operating principle and experimental verification

**Figure 2:**
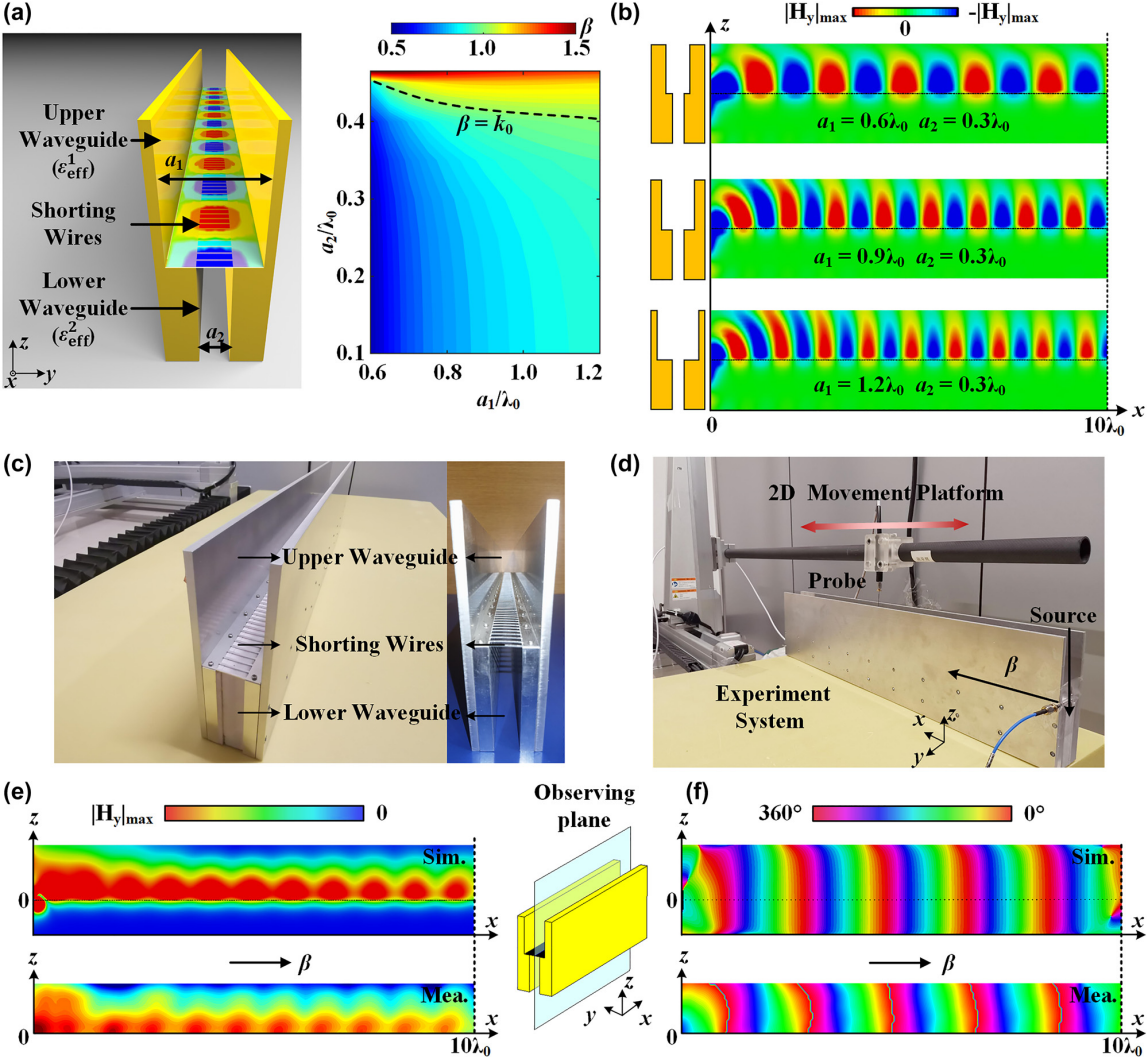
The theoretical analysis and experimental verification. (a) Structure and dispersion relationship of the stepped waveguide metamaterial. (b) Propagation in the stepped waveguide SPP under three groups of parameters. (c) Fabricated prototype and (d) experimental setup for the stepped waveguide metamaterial. Numerical and experimental (e) magnitude and (f) phase of the transverse magnetic field inside the stepped waveguide.

The structure of the stepped waveguide metamaterial is demonstrated in Figure 2a. This design includes an air-filled upper waveguide and lower waveguide with perfect electrical boundaries but different widths. By taking advantage of the waveguide effective plasmonics [30], we can define the effective material parameters of the TE_10_ mode in the waveguide to realize the effective permittivity as a plasmonic material. The field distribution of the TE_10_ mode is uniform along the height and sinusoidal across the width of the waveguide; thus, the effective permittivity hinges on the width of the waveguide. Specifically, the upper waveguide with a large width has a positive effective permittivity, while the lower waveguide has a negative effective permittivity, which is exactly the condition supporting regular SPPs on the interface between insulators with positive permittivity and plasmonic materials with negative permittivity. Therefore, the proposed stepped waveguide metamaterial could support an identical field distribution to regular SPPs. Shorting wires on the interface of the upper and lower waveguides are adopted to remove the degenerated TM mode, which has an operating frequency identical to that of the TE_10_ mode but presents an undesired field distribution in the stepped waveguide.

Figure 2a exhibits the propagating property of SPPs in the stepped waveguide metamaterials. Different from the SPP propagation on the two-dimensional interface between the plasmonic material and insulator, the step-shaped boundary in the stepped waveguide at the interface between the upper waveguide and lower waveguide introduces an extra surface impedance and constructs the dispersion relationship of the SPP propagation, which is derived in Supplementary Note 1. The dispersion relationship of the stepped waveguide can be written as follows:

$$\frac{\varepsilon_{\text{eff}}^{1}}{\sqrt{{\left(\frac{\beta_{\text{spp}}}{k_{0}}\right)}^{2}-\varepsilon_{\text{eff}}^{1}}}+\frac{\varepsilon_{\text{eff}}^{2}}{\sqrt{{\left(\frac{\beta_{\text{spp}}}{k_{0}}\right)}^{2}-\varepsilon_{\text{eff}}^{2}}}=\frac{j120\pi }{Z_{s}}$$

where $\varepsilon_{\text{eff}}^{1}$ and $\varepsilon_{\text{eff}}^{2}$ are the effective relative permittivity of the upper waveguide and lower waveguide derived from the waveguide effective plasmonics, *β*_spp_ is the propagating constant in the stepped waveguide, and *Z*_s_ is the surface impedance generated by the stepped waveguide metamaterial. This dispersion relationship can be simplified to the classic propagating constant for regular SPP as $\beta_{\text{spp}}=\sqrt{\frac{\varepsilon_{\text{eff}}^{1}\varepsilon_{\text{eff}}^{2}}{\varepsilon_{\text{eff}}^{1}+\varepsilon_{\text{eff}}^{2}}}$ when there exists no discontinuity in the boundary of the stepped waveguide, i.e., *Z*_s_ = ∞. The propagating constant varying with *a*_1_ and *a*_2_ is calculated and depicted in Figure 2a. Figure 2b shows the propagating characteristics in the stepped waveguide with three different groups of parameters. It is noted that the effective SPP in the stepped waveguide can propagate as an unusual fast wave (*β*_spp_ < *k*_0_), as Figure 2a shows, which is unattainable in a regular SPP using natural plasmonic materials. The fast-wave propagating property makes it possible to realize direct coupling and radiation between the stepped waveguide and free space, thus eliminating extra structures such as coupling rasters. As a result, surface plasmons in the stepped waveguide could be excited by a source such as a rectangle waveguide in free space instead of an attached small dipole in Figure 2. The detailed performances of direct coupling and radiation are discussed in Supplementary Note 2.

To validate the design concept of the stepped waveguide metamaterial and the field distributions of the effective SPP, we fabricate a prototype of the stepped waveguide metamaterial with aluminum, which is a nearly perfect electrical conductor at microwave frequencies. Limited by our experimental condition, we conduct the experiment at microwave frequency to verify the design concept and examine the exact field distribution in the stepped waveguide, and the loss issue of the metal at higher frequencies is discussed in the following section by numerical simulations. As demonstrated in Figure 2c, the widths of the upper and lower waveguides are 0.6*λ*_0_ and 0.3*λ*_0_ (*λ*_0_ is the wavelength in free space at the operating frequency) to realize positive and negative permittivity, respectively. To examine the effective SPP performance of the proposed design, the waves are excited by a small dipole moment from one end of the stepped waveguide. A two-dimensional (2-D) scanning movement platform is used to detect the field distribution inside the waveguide by a dipole probe, as shown in Figure 2d, with the detailed experimental setup introduced in the Methods. Limited by the scanning devices, only the region in the upper waveguide is scanned. As indicated in Figure 2e and f, the measured transverse magnetic field magnitude and phase of the effective SPP in the upper waveguide of the stepped waveguide are highly consistent with the numerical results, validating that the field distribution in the stepped waveguide is identical to that in a regular SPP.

### 2.2 Low-loss replica of regular SPP

**Figure 3: j_nanoph-2022-0810_fig_003:**
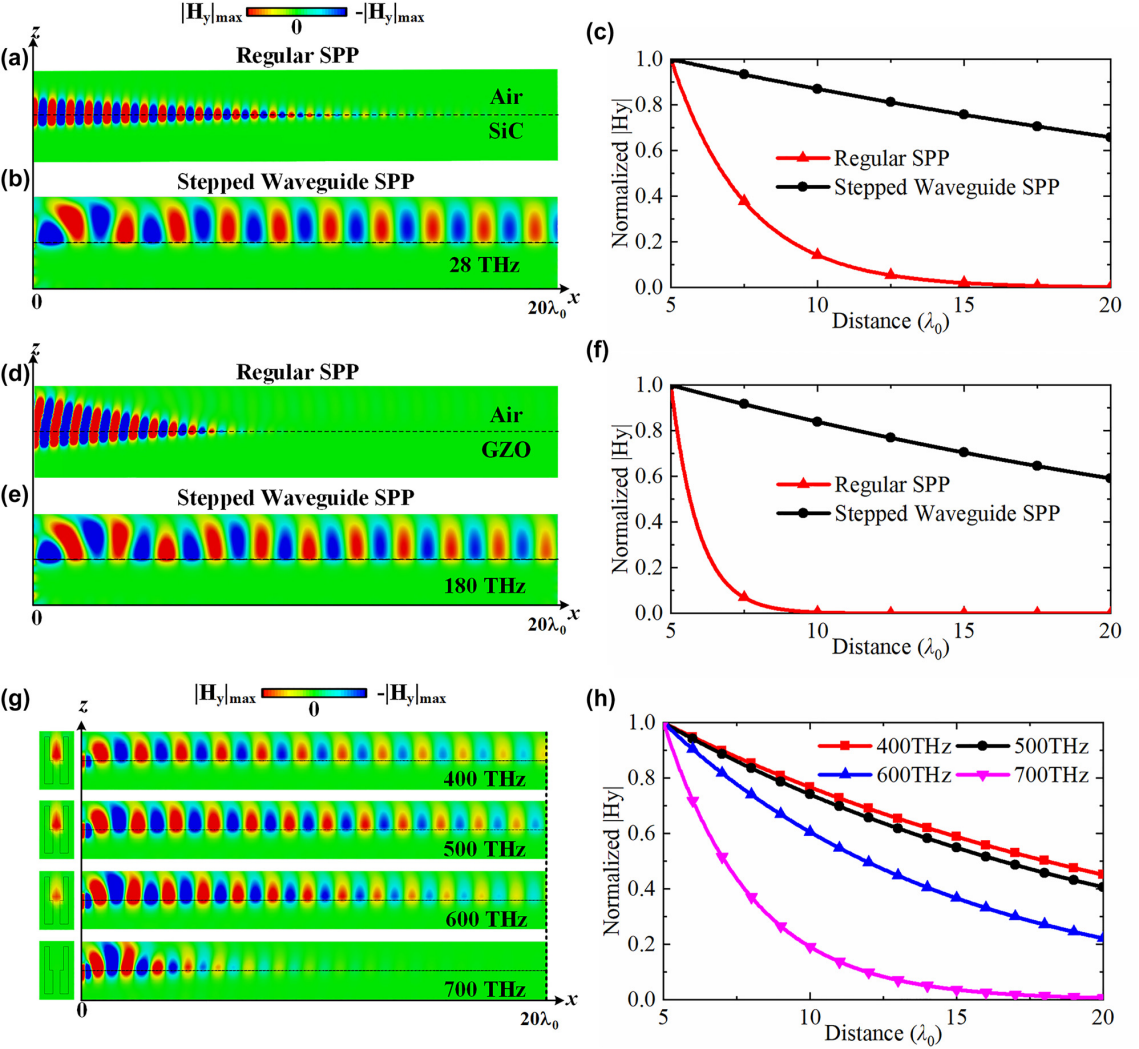
The low-loss property of the stepped waveguide. The magnitude comparison of transverse magnetic field distributions of regular SPP and stepped waveguide SPP at (a)–(c) 28 THz and (d)–(f) 180 THz. (g) Losses and (h) comparison of the stepped waveguide SPP at a higher frequency from 400 THz to 700 THz. The stepped waveguide in all frequencies has *a*_1_ = 0.6*λ*_0_, *a*_2_ = 0.3*λ*_0_, and a height of 2*λ*_0_.

As mentioned above, a regular SPP suffers from inherent losses in natural plasmonic materials. Compared to a regular SPP, stepped waveguide metamaterials can realize plasmonic properties and identical field distributions to regular SPPs without natural plasmonic materials and thus without involving the loss issue. In Figure 3, we have shown SPP propagation on a natural plasmonic material and then proposed a stepped waveguide metamaterial at different frequencies. In Figure 3a and b, a regular SPP is realized at the interface of air and silicon carbide (SiC) with a Lorentzian model [27]. SiC is a relatively low-loss plasmonic material with a relative permittivity of −1.95 + 0.165*j* at 28 THz. The boundary of the stepped waveguide metamaterial is silver, as described by the Drude model [27]. Figure 3c shows that the plasmonic propagation in the stepped waveguide SPP has a much lower loss than that in the regular SPP. The propagation lengths (distance after which the SPP filed intensity decreases to 1/*e* of its starting value) of the SPP in the natural plasmonic material and stepped waveguide are 27.44 µm (2.561*λ*_0_) and 384.76 µm (35.912*λ*_0_), respectively, which means a 14-fold improvement for the stepped versus regular SPP on an Air/SiC interface.

As another comparison example, in Figure 3d and e, gallium-doped zinc oxide (GZO) with a relative permittivity of −1.98 + 0.60*j* [36] is adopted to realize the regular SPP at 180 THz, and the material of the stepped waveguide metamaterial is still silver with the Drude model. The loss comparison between a regular SPP in a natural plasmonic material and an effective SPP in a stepped waveguide metamaterial is demonstrated in Figure 3f. Their propagation lengths are 1.56 µm (0.935*λ*_0_) and 47.59 µm (28.547*λ*_0_), respectively, representing a 30-fold improvement. The losses in this approach mainly come from the nonideal metal boundary composed of silver and gradually increase with increasing frequency. Figure 3g shows the transverse magnetic fields to demonstrate the loss issue in the stepped waveguide at higher frequencies. As shown in Figure 3h, the effective SPP in the stepped waveguide metamaterial can maintain reasonable low-loss propagation up to a near-infrared region lower than 600 THz. At higher frequencies above 700 THz, losses in silver terminate the SPP propagation. Based on these results, the proposed effective SPP method remains valid in visible light up to 600 THz, indicating a wide operating frequency range as a low-loss replica of a regular SPP.

## 3 Discussion

### 3.1 Interconnection with regular SPP

**Figure 4: j_nanoph-2022-0810_fig_004:**
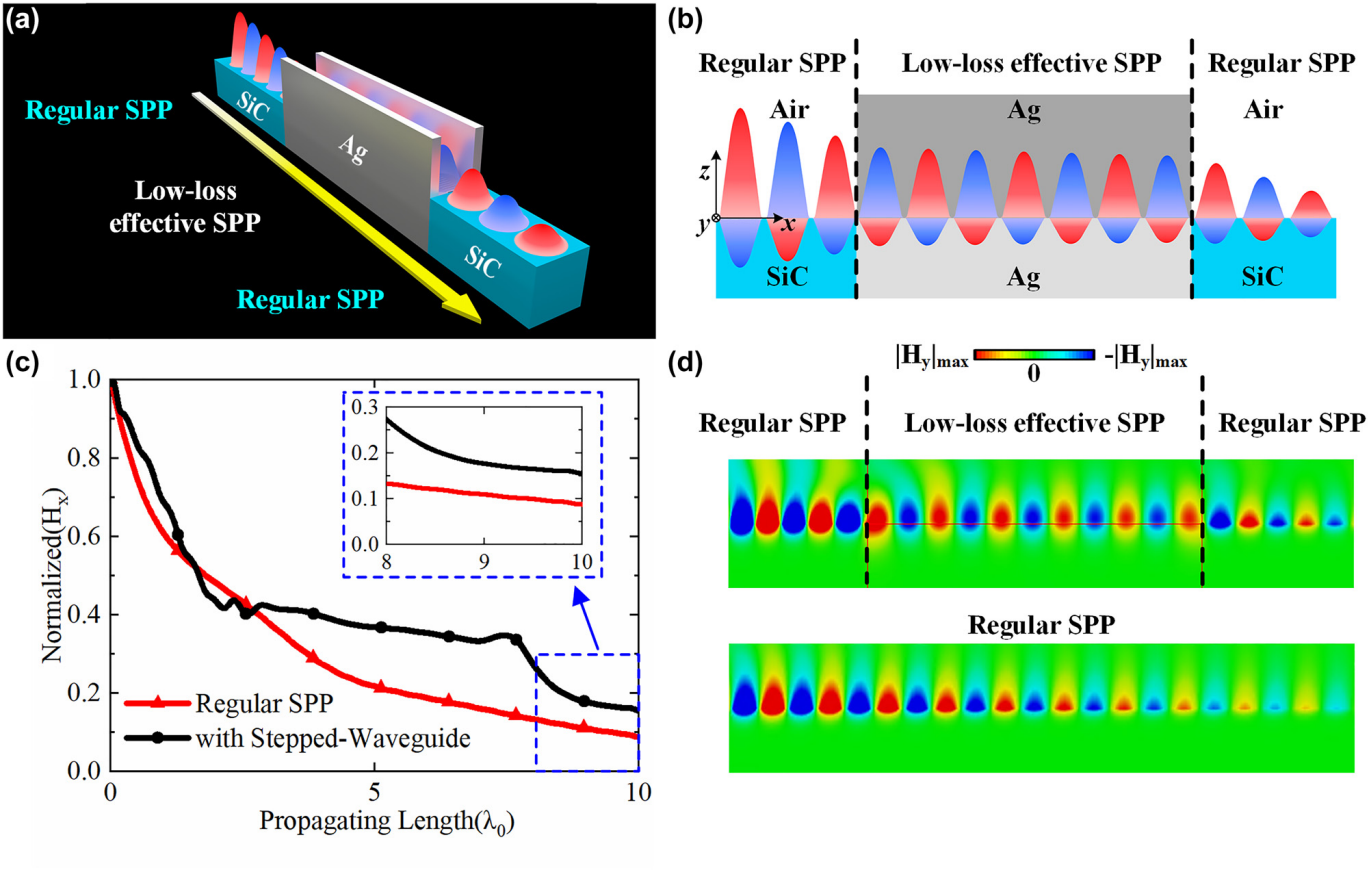
The low-loss interconnection and interaction with regular SPP. (a) Direct interconnection between regular SPP and stepped waveguide SPP. (b) Structure and field interaction of the interconnection. (c) Magnitude decreasing and (d) field distributions of the transverse magnetic field along the propagation in regular SPP and interconnections between regular SPP and stepped waveguide SPP. The width of the SiC is 1.5*λ*_0_, in the stepped waveguide, *a*_1_ = *λ*_0_, *a*_2_ = 0.42*λ*_0_, and the height is 2*λ*_0_.

In spoof SPP designs in the literature, there are no natural plasmonic materials, thus showing low-loss plasmonic properties at microwave frequencies. In contrast, the stepped waveguide metamaterial constructs two uniform regions with both positive and negative permittivity reproducing an identical field distribution and propagating property of a regular SPP, which has been verified in Figure 2e and f. This consistency leads to significant compatibility between regular SPPs and effective SPPs in a stepped waveguide, making it possible for the effective SPP in the stepped waveguide to behave as a replica of a regular SPP. In the example shown in Figure 4a, the effective SPP in the stepped waveguide can directly interconnect with the regular SPP. In Figure 4b, the regular SPP on the interface between SiC and air can directly propagate to the interface of the upper waveguide and lower waveguide in the stepped waveguide and finally return to the regular SPP. As a merit of the stepped waveguide, SPP losses are significantly reduced by replacing a length of a regular SPP with an effective SPP in the stepped waveguide, as shown in Figure 4c. The magnitude loss of the stepped waveguide is approximately 1.47 dB in a propagation length of 5*λ*_0_, which is much lower than the value of approximately 9.40 dB in a regular SPP with natural plasmonic materials. Figure 4d exhibits the transverse magnetic field distribution in a regular SPP and a stepped waveguide metamaterial. As the regions with positive permittivity and negative permittivity in the regular SPP and stepped waveguide are seamlessly connected, the identical field distribution accomplishes a smooth propagation transition and continuous field interactions for SPP waves. This low-loss property and intrinsic compatibility could lead to versatile low-loss SPP devices. On the other hand, compared with other waveguide-based effective SPP [37–40], the stepped waveguide metamaterial with only air medium realizes a complete SPP structure leveraging the structure flexibility of both upper and lower waveguides for arbitrary effective permittivities in the two regions. This structure not only avoids dielectric losses but also guarantees the convenient realization of SPP property without consideration of material choice and frequency restriction, indicating high-level freedom in a simple structure for versatile and feasible plasmonic applications.

### 3.2 Low-loss SPP device applications

**Figure 5:**
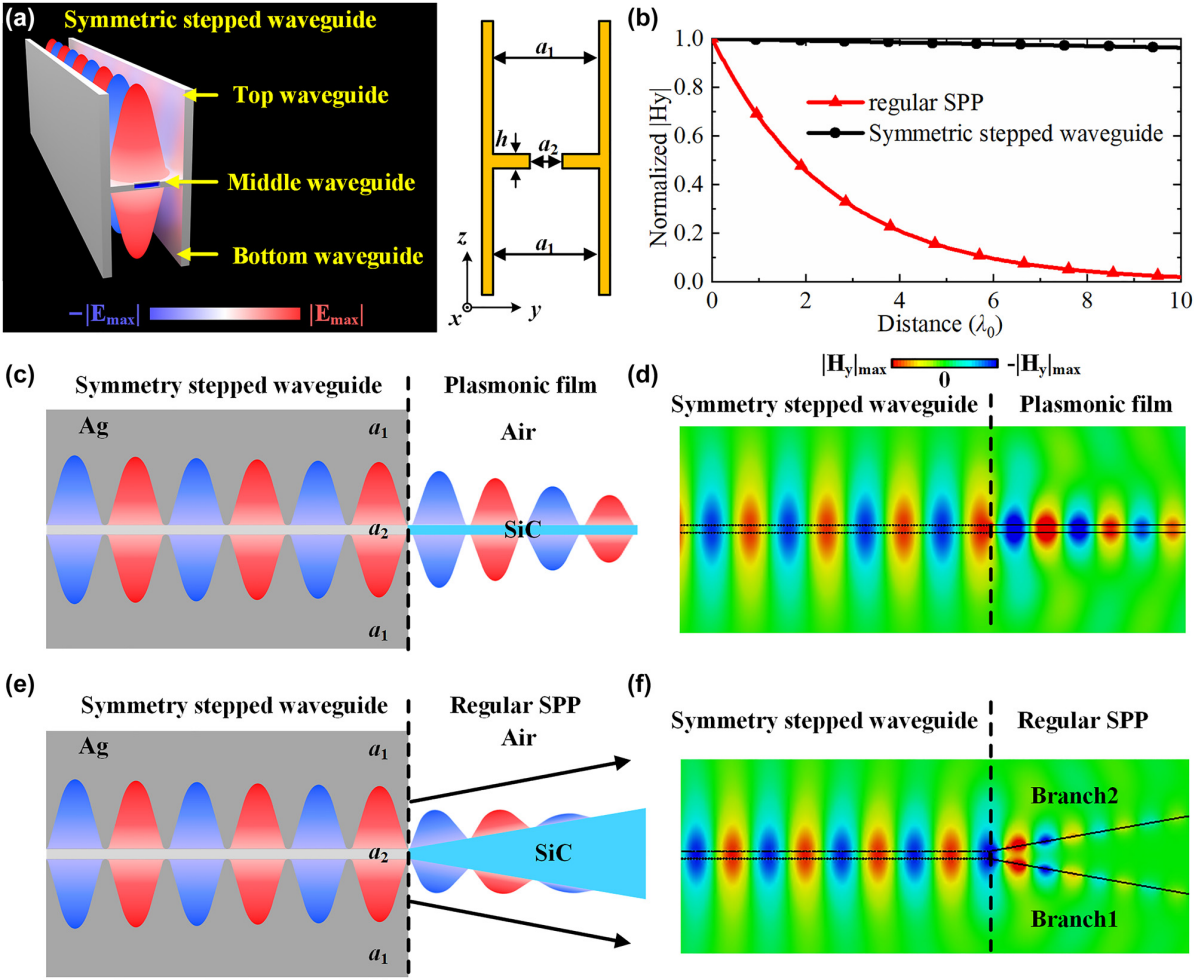
Applications of stepped waveguide metamaterial. (a) Structure of the symmetric stepped waveguide. (b) Magnitude comparison of transverse magnetic field distributions of regular SPP and symmetric stepped waveguide SPP. We have *a*_1_ = 1.4*λ*_0_, *a*_2_ = 0.4*λ*_0_, and *h* = 0.1*λ*_0_. (c)–(d) Direct interconnection and interaction between a symmetric stepped waveguide and a plasmonic film. We have *a*_1_ = 1.4*λ*_0_, *a*_2_ = 0.4*λ*_0_, and *h* = 0.1*λ*_0_. (e)–(f) Design and magnitude of the transverse magnetic field of the SPP power divider. We have *a*_1_ = 1.4*λ*_0_, *a*_2_ = 0.42*λ*_0_, and *h* = 0.1*λ*_0_.

Manipulating an SPP on a plasmonic film is another useful method to realize a relatively low-loss SPP propagation [41, 42]. Benefiting from the stepped waveguide metamaterial, we can also realize a low-loss effective SPP in a symmetric stepped waveguide by properly designing the stepped waveguide metamaterials. As Figure 5a demonstrates, a structure with two symmetric “step” layers in the waveguide is adopted to realize two interfaces between positive permittivity and negative permittivity, which exactly imitates the structure of the plasmonic film. Specifically, the proposed structure is divided into three regions. The top and bottom waveguides have larger widths behaving as positive effective permittivity, while the middle waveguide has a smaller width behaving as negative effective permittivity. These two interfaces successfully constitute the effective SPP in this symmetric stepped waveguide collectively. As shown in Figure 5b, the symmetric stepped waveguide further improves the propagation length of the effective SPP to 2.872 mm (268.086*λ*_0_) at 28 THz, which is more than a hundred-fold improvement compared to a regular SPP on an Air/SiC interface at 28 THz. At the wavelength of 1550 nm (193.5 THz), the symmetric stepped waveguide still holds the low-loss property and the propagation length of the effective SPP is 225.53 µm (145.503*λ*_0_), which is larger than those in regular SPP waveguide design with traditional plasmonic materials [31–35], as exhibited in Table 1. The property of symmetric stepped waveguide at 1550 nm is displayed in Supplementary Note 3. Similarly, an effective SPP in a symmetric stepped waveguide also presents an identical field distribution to a regular SPP on the plasmonic film, realizing direct interconnection and interaction with the regular SPP on the plasmonic film, which is exhibited in Figure 5c and d. Meanwhile, it is possible to realize an asymmetric SPP mode in an asymmetric stepped waveguide; the details of the asymmetric stepped waveguide are presented in Supplementary Note 4. In addition, an SPP power divider is developed for this technique. As shown in Figure 5e, a wedge-shaped plasmonic material (SiC) is directly connected at the end of the symmetric stepped waveguide. This SiC wedge is designed with an angle of 20° to guide the two paths of surface waves on the two interfaces of the symmetric stepped waveguide into two SPP branches, which can propagate a regular SPP on the Air/SiC interface and realize the power divider. As demonstrated in Figure 5f, the transverse magnetic field distributions of the proposed SPP power divider verify its feasibility to split the SPP energy into two branches, which is not easy to realize using regular SPP techniques.

**Table 1:** Comparison of the low-loss SPP at 1550 nm.

Literature	Operating wavelength	Attenuation coefficient	Propagation length
[31]	1550 nm	0.047 dB/μm	185.56 μm
[32]	1550 nm	∼0.2 dB/μm	∼43.43 μm
[33]	1550 nm	0.138 dB/μm	63 μm
[34]	1550 nm	0.217 dB/μm	40 μm
[35]	1550 nm	0.2 dB/μm	43.43 μm
Proposed	1550 nm	0.038 dB/μm	225.53 μm

## 4 Conclusions

In this work, we have theoretically and experimentally demonstrated the concept of a stepped waveguide metamaterial as a low-loss effective replica of an SPP. Taking advantage of the structural dispersion, the stepped waveguide realizes two regions with positive and negative permittivity to reproduce the interface between the plasmonic material and insulator for surface plasmon waves. With this property, the effective SPP in stepped waveguide metamaterial presents the same field distribution as that of a regular SPP on natural plasmonic material and enables seamless low-loss interconnection and interaction with the regular SPP. Without using lossy plasmonic materials, the stepped waveguide realizes a low-loss replica of SPP with a propagation length improved by 1–2 orders of magnitude compared to a regular SPP and outperforms regular low-loss SPP waveguide designs with traditional plasmonic materials. The stepped waveguide metamaterials remove the limit of materials and employ only air and metal boundaries to realize arbitrary plasmonic material properties. Based on the fundamental surface plasmon waves in stepped waveguide, we could realize an effective SPP in a symmetric stepped waveguide to design an SPP power divider. With the advantages above, the stepped waveguide metamaterial can provide a low-loss replica of an SPP and offer new paths to low-loss SPP devices in plasmonics and nanophotonics.

## 5 Materials and methods

### 5.1 Numerical simulations

The numerical data in Figure 2a were obtained with the commercial software of MATLAB. The numerically simulated results in Figures 2 [Fig j_nanoph-2022-0810_fig_003] [Fig j_nanoph-2022-0810_fig_004]–5 were performed by full-wave simulations using the commercial software CST STUDIO SUITE^®^. In the simulation setup, a time-domain solver with hexahedral meshing was adopted. The cells per max model box edge were set to 20. The boundary of the simulated region is set to be open boundaries in all directions to mimic an open system in free space. The excitation of the stepped waveguide in Figures 2 [Fig j_nanoph-2022-0810_fig_003] [Fig j_nanoph-2022-0810_fig_004]–5 is a discrete port perpendicular to the interface at one end of the stepped waveguide with a height of *λ*_0_/50. The excitations for results in Figure 4 is a natural plasmonic SPP with a waveguide port in the correct port mode connecting to the stepped waveguide. The dimensions of the shorting wires in Figures 3 [Fig j_nanoph-2022-0810_fig_004]–5 are 0.005*λ*_0_ in height and 0.02*λ*_0_ in width. The Drude model of silver is set to be $\varepsilon_{Ag}=\varepsilon_{\infty }-\omega_{p}^{2}/\omega \left(\omega +i\gamma \right)$ , where *ε*_∞_ = 5, *ω*_p_ = 1.37 × 10^16^ rad/s, and *γ* = 2.73 × 10^13^ rad/s.

### 5.2 Experiment setup

The prototype of the stepped waveguide metamaterial presented in Figure 2c and d is fabricated totally with aluminum. The upper waveguide has a width of 36 mm (0.6*λ*_0_) and the lower waveguide has a width of 18 mm (0.3*λ*_0_) at the operating frequency of 5 GHz (*λ*_0_ = 60 mm). The shorting wires have a width of 1.2 mm (0.02*λ*_0_) and a height of 0.8 mm (0.0133*λ*_0_), positioned with a period of 6 mm (0.1*λ*_0_). The SPP in the stepped waveguide is excited by a small dipole at one end of the stepped waveguide, and the field distributions are detected via the probe on the 2-D scanning movement platform. The magnitude and phase of the field distributions are calculated from the transmission coefficient between the excitation and detector obtained by the vector network analyzer.

## Supplementary Material

Supplementary Material Details
