# Supplementary material for: Stepped waveguide metamaterials as low-loss effective replica of surface plasmon polaritons
Source: Nanophotonics. 2023 Mar 1;12(7):1285–93. doi: 10.1515/nanoph-2022-0810 (PMC11636075; doi:10.1515/nanoph-2022-0810)
Supplement: Supplementary file 1 — Supplementary Material Details [file j_nanoph-2022-0810_suppl_001.docx]

**Supplementary Materials for**

***Stepped Waveguide Metamaterials as Low-Loss Effective Replica of Surface Plasmon Polaritons***

Xu Qin^1^*, Yijing He^2^*, Wangyu Sun^1^*, Pengyu Fu^1^, Shuyu Wang^1^, Ziheng Zhou^3^ and Yue Li^1,4+^

*^1^Department of Electronic Engineering, Tsinghua University, Beijing 100084, China*

*^2^School of Integrated Circuits and Electronics, Beijing Institute of Technology, Beijing 100081, China.*

*^3^College of Physics and Information Engineering, Fuzhou University, Fuzhou 350108, China*

*^4^Beijing National Research Center for Information Science and Technology, Beijing 100084, China.*

*These authors contributed equally to this work.

^+^ lyee@tsinghua.edu.cn

**Supplementary Note 1.** Propagation constant in stepped waveguide.

**Figure S1.** Theory of the effective circuit model in stepped waveguide.

**Figure S2.** Effective SPP propagation in stepped waveguide with effective permittivity.

**Supplementary Note 2.** Direct radiation and coupling of the stepped waveguide.

**Figure S3.** Direct radiation and coupling of the stepped waveguide.

**Supplementary Note 3.** SPP in symmetric stepped waveguide at 1550nm.

**Figure S4.** Normalized magnetic field in symmetric stepped waveguide at 1550nm.

**Supplementary Note 4.** SPP mode in asymmetric stepped waveguide

**Figure S5.** Structure and field distribution of the asymmetric stepped waveguide.

**Supplementary Note 1.** Propagation constant in stepped waveguide.


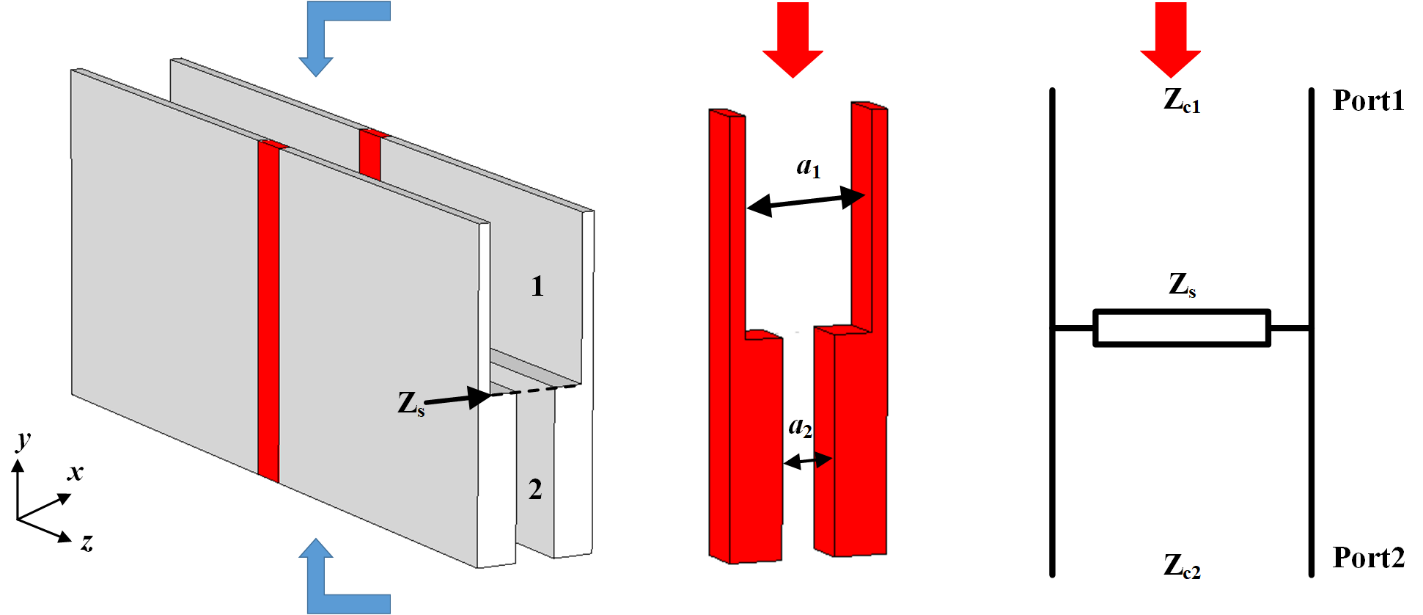


**Figure S1. Theory of the effective circuit model in stepped waveguide.** The stepped waveguide is analyzed with a circuit model to derive the additional impedance of the discontinuity in stepped waveguide.

As shown in Figure S1, a small length of the stepped waveguide is extracted to analyze the circuit model of the stepped structure. If we cover the extracted stepped structure by PEC from +z and –z direction, the stepped structure is transformed into a discontinuous waveguide propagating along y direction. Here, we could establish a circuit model of the waveguide in TE_10_ mode with z polarization, as shown in Figure S1, where *Z*_c1_ and *Z*_c2_ are the characteristic impedance of the wider and narrower waveguide with width of *a*_1_ and *a*_2_, *Z*_s_ is the effective parallel impedance generated by the discontinuity in the waveguide. We could derive Z_c1_ and Z_c2_ by:

$$Z_{c}=\sqrt{\frac{\mu_{0}}{\varepsilon_{eff}}}=\sqrt{\frac{\mu_{0}}{\varepsilon_{0}-4a^{2}\text{/}\lambda_{0}^{2}}}$$

where *a* is the width of the waveguide, *λ*_0_ is the operating wavelength in free space, $\varepsilon_{eff}=\varepsilon_{0}-4a^{2}/\lambda^{2}$ is the effective permittivity of the TE10 mode in the waveguide, *μ*_0_ and *ε*_0_ is the permittivity and permeability in free space, respectively. By reflection coefficient S_11_ of Port 1, we could derive *Z*_s_ by:

$$Z_{s}=\frac{Z_{c1}Z_{c2}(S_{11}+1)}{Z_{c2}(1-S_{11})-Z_{c1}(1+S_{11})}$$

It is noted that the shorting wires in the stepped waveguide has little influence in the circuit model or the propagation property of the effective SPP in stepped waveguide, thus the shorting wires have not been discussed in detail. By full-wave simulations, we have derived the *Z*_s_ in the circuit model of the stepped waveguide with different parameters. In the stepped waveguide, *Z*_s_ generated from the discontinuity could be treated as a surface impedance in the interface between the wider waveguide and the narrower waveguide.


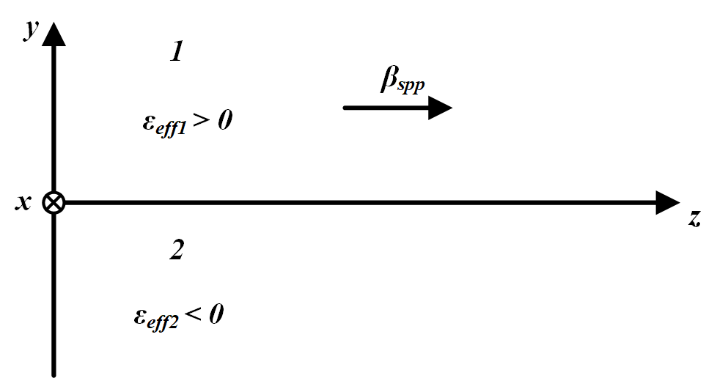


**Figure S2. Effective SPP propagation in stepped waveguide with effective permittivity.**

Then we consider an effective SPP propagation on the interface with a surface impedance of *Z*_s_, as shown in Figure S2. We could write the fields as follows:

$$\begin{matrix} H_{x}^{1}=H_{1}e^{-j\beta_{spp}z}e^{-\alpha_{1}y} \\ H_{x}^{2}=H_{2}e^{-j\beta_{spp}z}e^{\alpha_{2}y} \\ E_{z}^{1}=\frac{\alpha_{1}}{j\omega\varepsilon_{eff}^{1}\varepsilon_{0}}H_{1}e^{-j\beta_{spp}z}e^{-\alpha_{1}y} \\ E_{z}^{2}=-\frac{\alpha_{2}}{j\omega\varepsilon_{eff}^{2}\varepsilon_{0}}H_{2}e^{-j\beta_{spp}z}e^{\alpha_{2}y} \end{matrix}$$

where $\alpha_{1}=\sqrt{\beta_{spp}^{2}-k_{1}^{2}}$,$\alpha_{2}=\sqrt{\beta_{spp}^{2}-k_{2}^{2}}$are the attenuation coefficient in two regions at y direction, respectively, and *k*_1_,*k*_2_ are the propagating constants for TEM wave in region 1 and region 2. Then we can derive the reactance $Y_{y}^{1}$ and $Y_{y}^{2}$ from the electric and magnetic fields by $Y_{y}=H_{x}/E_{z}$. Considering the surface impedance of *Z*_s_ parallel in the circuit model, the effective SPP propagation would satisfy the impedance matching $Y_{y}^{1}+Y_{s}=Y_{y}^{2}$,which could be written as follows:

$$\frac{\varepsilon_{eff}^{1}}{\sqrt{(\frac{\beta_{spp}}{k_{0}})^{2}-\varepsilon_{eff}^{1}}}+\frac{\varepsilon_{eff}^{2}}{\sqrt{(\frac{\beta_{spp}}{k_{0}})^{2}-\varepsilon_{eff}^{2}}}=\frac{j120\pi}{Z_{s}}$$

where *k*_0_ is the propagation constant in free space. In fact, the surface impedance in the stepped waveguide could be treated as a parallel inductor, which means the right side of Equation S4 is a positive real number and the propagation constant *β*_spp_ have real solutions to realize effective SPP in the stepped waveguide.

**Supplementary Note 2. Direct radiation and coupling of the stepped waveguide.**


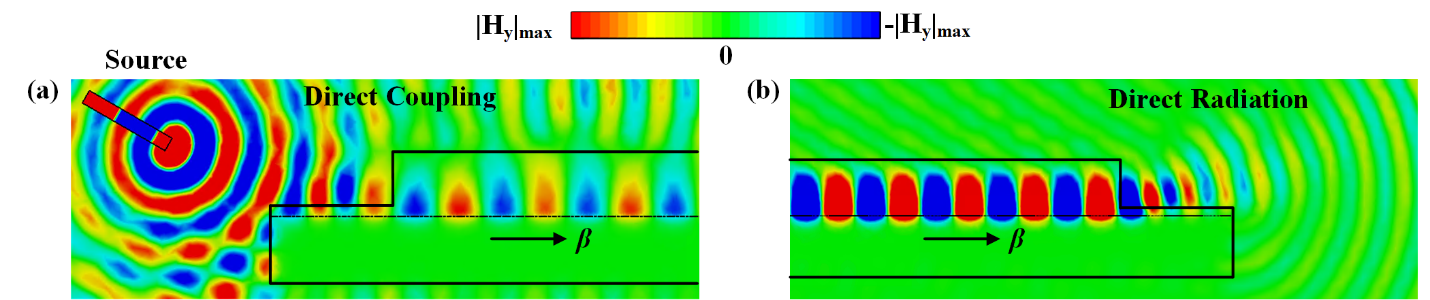


**Figure S3. Direct radiation and coupling of the stepped waveguide.**

In Figure S3, the stepped waveguide is constructed with a perfect electric conductor with *a*_1_=0.6*λ*_0_, *a*_2_=0.3*λ*_0_. In this setup, the effective SPP propagation in the stepped waveguide behaves as a fast wave, while regular SPP usually behaves as a slow wave. This property makes it possible for the effective SPP in the stepped waveguide to realize direct radiation and coupling to free space. In Figure S3a, the effective SPP in the stepped waveguide is excited by the direct coupling of the source in free space. The source is a rectangular waveguide with TE_10_ mode, and a part with a height of 1.2*λ*_0_ and length of 2*λ*_0_ has been cut off from the stepped waveguide with a total height of 3*λ*_0_ for better coupling performance. It could be seen that the effective SPP has been successfully excited through direct coupling from the rectangular waveguide in free space. In Figure S3b, similarly, a part with a height of 0.95*λ*_0_ and a length of 3*λ*_0_ has been cut off from the stepped waveguide with a total height of 2.5*λ*_0_ to realize direct radiation from the effective SPP to the free space. These two results have validated the direct coupling and radiation for the stepped waveguide.

**Supplementary Note 3.** SPP in symmetric stepped waveguide at 1550nm.


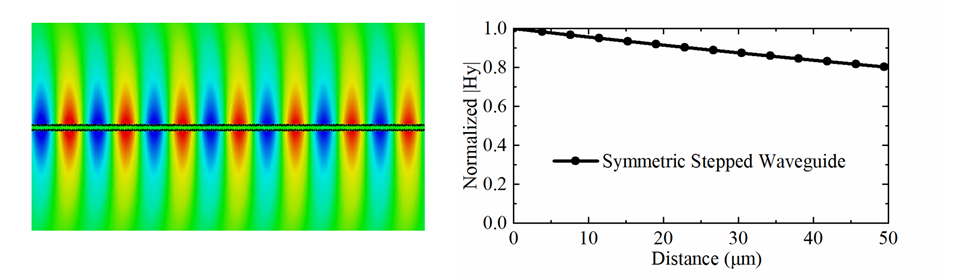


**Figure S4.** Normalized magnetic field in symmetric stepped waveguide at 1550nm.

In Figure S4, there exhibits the normalized magnetic field along the propagation of the SPP. The SPP propagation has a propagation length of 225.53μm (145.503λ_0_). In the structure of the symmetric stepped waveguide in Figure S4, we have a_1_=1.4 λ_0_, a_2_=0.3 λ_0_, and h=0.1 λ_0_. The dimensions of the shorting wires are 0.005 λ_0_ in height and 0.02 λ_0_ in width. The Drude model of silver is set to be $\varepsilon_{Ag}=\varepsilon_{\infty}-\omega_{p}^{2}/\omega(\omega+i\gamma)$ , where ε_∞_ = 5, ω_p_ = 1.37×10^16^ rad/s, γ = 2.73×10^13^ rad/s.

**Supplementary Note 4.** SPP mode in asymmetric stepped waveguide


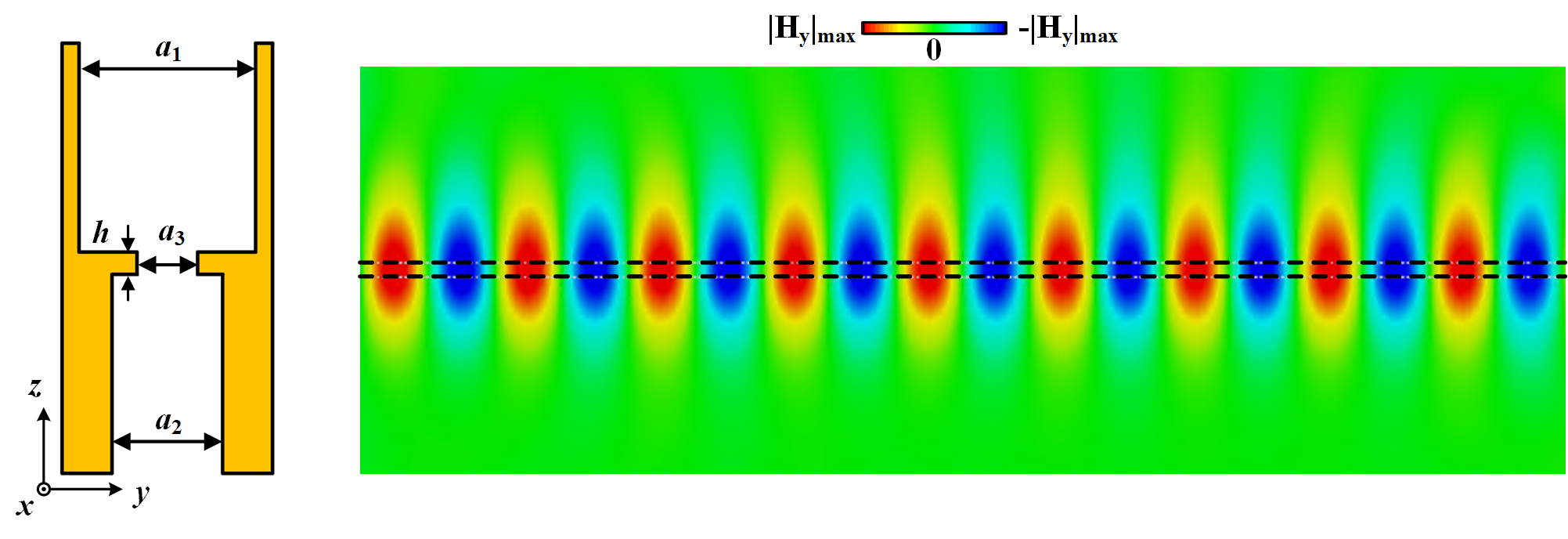


**Figure S5.** Structure and field distribution of the asymmetric stepped waveguide.

As shown in Figure S5, the symmetric stepped waveguide could be expanded to an asymmetric mode of SPP by adopting different widths in the top waveguide and bottom waveguide. Accordingly, the surface plasmons in the asymmetric stepped waveguide behave as an asymmetric SPP mode. To be specific, the rate of evanescence from the interface is higher in the narrower waveguide because of the smaller effective permittivity. Here the widths of the two waveguides are a_1_=1.4 λ_0_, a_2_=0.45 λ_0_, a_3_=0.8 λ_0_, and h=0.1 λ_0_, and the numerical simulation is conducted at 28 THz with silver.
